# Phytol-loaded soybean oil nanoemulsion as a promising alternative against *Leishmania amazonensis*

**DOI:** 10.3762/bjnano.16.126

**Published:** 2025-10-21

**Authors:** Victória Louise Pinto Freire, Mariana Farias Alves-Silva, Johny W de Freitas Oliveira, Matheus de Freitas Fernandes-Pedrosa, Alianda Maira Cornélio, Marcelo de Souza-Silva, Thayse Silva Medeiros, Arnóbio Antônio da Silva Junior

**Affiliations:** 1 Laboratory of Pharmaceutical Technology and Biotechnology, Department of Pharmacy, Federal University of Rio Grande do Norte-UFRN, Natal, RN, Brazil; 2 Immunoparasitology Laboratory, Department of Clinical and Toxicological Analysis, Centre of Health Sciences, Federal University of Rio Grande do Norte-UFRN, Natal, RN, Brazil; 3 Department of Morphology, Federal University of Rio Grande do Norte-UFRN, Natal, RN, Brazil

**Keywords:** cutaneous leishmaniasis, *Leishmania amazonensis*, nanoemulsion, negleted tropical disease, phytol

## Abstract

Leishmaniasis, caused by protozoa of the genus *Leishmania* spp., is a neglected tropical disease that poses a significant challenge to the public health in tropical and subtropical regions, affecting mainly low-income individuals. Current therapies are limited due to severe adverse reactions to currently available drugs, high cost, low patient adherence, and even the emergence of resistant strains. Examining safer and more effective alternatives, natural compounds such as phytol – a diterpene derived from chlorophyll – have attracted attention due to their broad biological activities. To increase their solubility, stability, and cell delivery, nanotechnology-based systems, such as nanoemulsions (NEs), represent a promising approach. In this study, soybean oil nanoemulsions loaded with phytol (PHYT-NE) were developed using the phase inversion composition (PIC) method, and then characterized and evaluated. The PHYT-NE had a mean droplet diameter close to 200 nm, a polydispersity index of less than 0.2, spherical shape, and a pH value compatible with cutaneous application. The formulation showed high colloidal stability for at least 30 days of storage and at least 15 days even under stress conditions, with no signs of macroscopic instability or changes in droplet size. The cytocompatibility of NEs was confirmed in 3T3 fibroblasts at the concentrations tested, indicating potential safety for in vivo trials. Notably, PHYT-NE exhibited significant time-dependent leishmanicidal activity against *Leishmania amazonensis* promastigotes, with lower IC_50_ values (up to five times lower at 48 hours) and up to 75% parasite death after 48 hours, showing greater antiparasitic activity compared to that of free phytol. Although the use of promastigotes represents a limitation, this model was used as a proof-of-concept, with promising evidence of the potential of PHYT-NE. Future studies in macrophage models infected with intracellular amastigotes will be essential to confirm the observed efficacy and validate the potential of PHYT-NE as a safe and effective topical therapy for cutaneous leishmaniasis.

## Introduction

Leishmaniasis is one of the 20 listed neglected tropical diseases (NTDs), affecting over 350 million people globally, with an alarming 700,000 to 1 million new cases reported annually [1–3]. It is caused by protozoan parasites of the genus *Leishmania* spp., and transmitted through the bite of infected female phlebotomine sandflies. During its life cycle, the parasite exhibits two main morphological forms: the promastigote, which resides in the insect vector and represents the infective stage, and the amastigote, the intracellular form found in vertebrate hosts [4–5].

Different *Leishmania* spp. species are responsible for distinct clinical manifestations, including (i) cutaneous leishmaniasis, (ii) mucocutaneous leishmaniasis, and (iii) visceral leishmaniasis. In cutaneous leishmaniasis, the infection triggers an immune-inflammatory cascade that produces painless ulcerative lesions. Depending on the immune status of the host and the infecting species, these lesions can progress to extensive tissue damage (i.e., from the cutaneous to the mucocutaneous form [6], particularly involving the nasal septum and ears) resulting in scarring, anatomical disfigurement, and consequent social stigmatization [7–9].

Current therapeutic strategies for leishmaniasis rely on pentavalent antimonial compounds (first-line therapy), amphotericin B, miltefosine, and paromomycin. Although these drugs are effective, their use is often limited by serious adverse effects such as cardiotoxicity, nephrotoxicity, hepatotoxicity, pancreatic toxicity, and teratogenicity. Moreover, resistance to antileishmanial agents, particularly pentavalent antimonials, has been increasingly reported [10–11].

As a result, plant-derived natural compounds have been extensively investigated as alternative therapeutic agents [12]. There are currently no herbal medicines used in the treatment of cutaneous leishmaniasis. However, previous studies have reported that diterpenes exhibit promising antileishmanial activity while displaying low toxicity to host cells [13–15]. One such compound is a phytol, a highly lipophilic, acyclic monounsaturated diterpene alcohol derived from chlorophyll metabolism in plants [16], and has demonstrated promising antileishmanial potential. For example, da Silva and colleagues (2015) [17] showed that a phytol-rich fraction extracted from *Lacistema pubescens* exhibited potent activity against *Leishmania amazonensis* promastigotes and intracellular amastigotes. However, the high lipophilicity of the phytol significantly limits its pharmaceutical application by reducing bioavailability [18]. To overcome these limitations, the incorporation of phytol into nanostructured delivery systems, has been proposed to improve its solubility, stability, and intracellular delivery efficiency [19].

Nanotechnology-based drug delivery systems that encapsulate bioactive molecules have proven effective against trypanosomatids [20–21] especially *Leishmania* spp. [22–23]. Among these systems, nanoemulsions (NEs) are one of the most common types. They are colloidal dispersions of two immiscible liquids – typically oil and water – stabilized by emulsifying agents such as surfactants and co-surfactants, forming a kinetically stable system. With droplet sizes ranging from 20 to 500 nm, NEs can significantly enhance drug permeability and bioavailability [24–27].

Therefore, the present study aimed to develop a nanoemulsion containing phytol, produced via the low-energy emulsification method, as a novel potential pharmacological alternative for the treatment of cutaneous leishmaniasis.

## Results

### Phytol-loaded soybean-oil nanoemulsion

**Figure 1:**
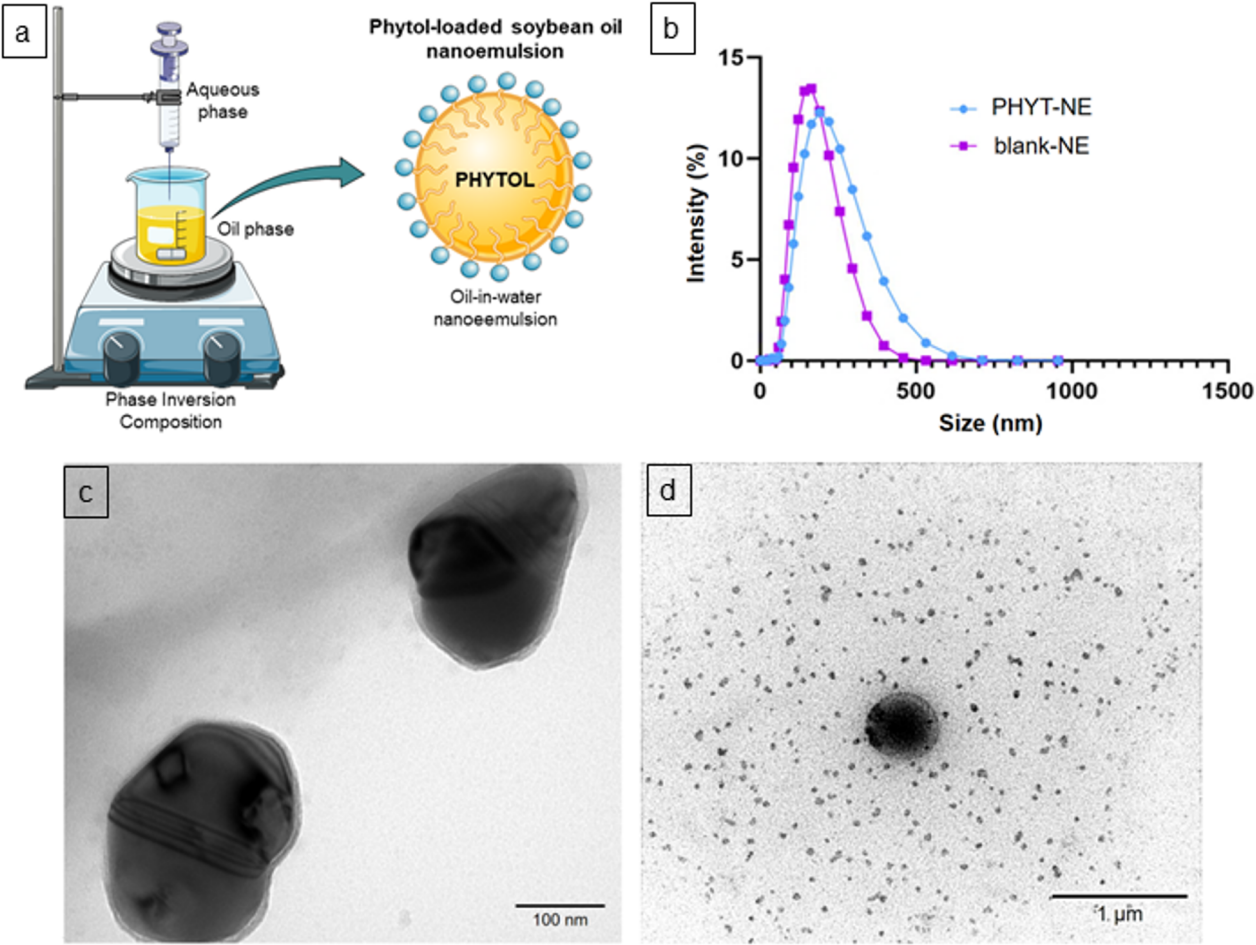
Schematic illustration of PHYT-NE preparation: (a) size distribution of nanodroplets (b) and morphological aspects of blank-NE (c) and PHYT-NE (d). Figure 1a) is adapted from Servier Medical Art (https://smart.servier.com/), licensed under CC BY 4.0 (https://creativecommons.org/licenses/by/4.0/).

Blank-NE and soybean oil nanoemulsions loaded with phytol (PHYT-NE) were successfully prepared using the phase inversion composition (PIC) method (Figure 1a), and exhibited droplet diameters of approximately 140 and 210 nm, respectively. An increase in droplet size was observed upon PHYT incorporation (*p* < 0.05). The polydispersity index (PdI) of the samples was below 0.2, indicating a monodisperse distribution, as shown in the size distribution graph (Figure 1b). Moreover, the zeta potential (ZP) of both samples was around −20 mV, with no significant changes upon phytol loading.

To evaluate the shape and morphological characteristics of the nanoemulsions, transmission electron microscopy (TEM) analysis was performed. The results revealed nanodroplets with spherical and oval shapes and a homogeneous size distribution, with diameters up to 250 nm, consistent with the dynamic light scattering (DLS) data (Figure 1c,d).

### Colloidal and physicochemical stability

The colloidal and physicochemical stability of the NEs was evaluated over a period of 30 days by monitoring droplet size, PdI, zeta potential, and pH values. In parallel, the samples were subjected to centrifugation at two different speeds (960 and 8600*g*) to simulate stress conditions. After centrifugation, droplet size, PdI, and ZP were assessed over a period of up to 15 days.

**Figure 2:**
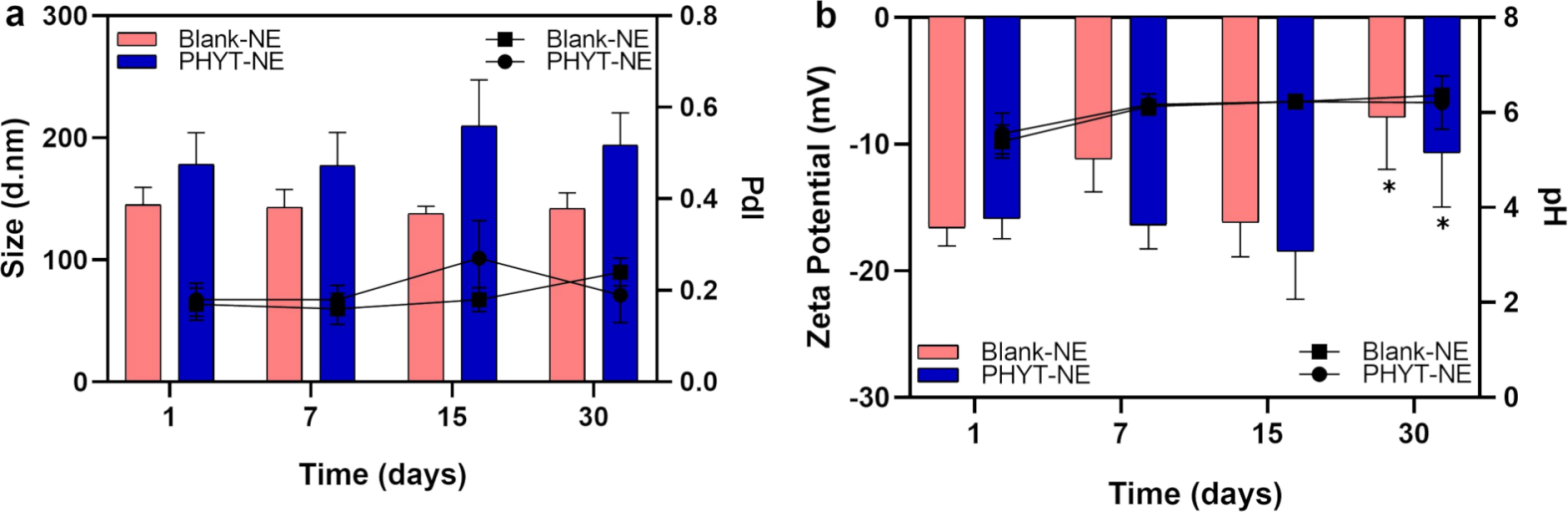
Colloidal and physicochemical stability of nanoemulsions regarding size, PdI (a), zeta potential and pH (b) over 30 days.

At the end of the 30-day stability study, both blank-NE and PHYT-NE remained stable with no changes in their initial macroscopic appearance (opaque, white, milky, and with low viscosity) and remained unchanged even after centrifugation. Among the physicochemical parameters evaluated, only the zeta potential showed a significant reduction at day 30 (*p* < 0.05), while no significant changes were observed in the other parameters, as shown in Figure 2a,b. Similarly, the formulations maintained their stability under all centrifugation conditions and time points analyzed, as presented in Table 1.

**Table 1:** Physicochemical properties of nanoemulsions after centrifugation stability assessment over 15 days.

Samples	Rotation (*g*)	Time (days)	Size (nm) ± SD	PdI ± SD	ZP (mV) ± SD

blank-NE	960	7	143.2 ± 3.27	0.19 ± 0.022	−18.4 ± 0.72
	8600	7	142.6 ± 3.15	0.16 ± 0.025	−18.2 ± 0.64
	960	15	145.8 ± 1.90	0.18 ± 0.019	−19.5 ± 0.58
	8600	15	144.5 ± 1.30	0.15 ± 0.010	−19.6 ± 0.61

PHYT-NE	960	7	208.5 ± 2.45	0.18 ± 0.027	−19.1 ± 0.58
	8600	7	205.2 ± 2.25	0.15 ± 0.041	−19.5 ± 0.52
	960	15	218.5 ± 1,29	0.19 ± 0,008	−24.2 ± 0.61
	8600	15	203.1 ± 1.12	0.13 ± 0.020	−21.9 ± 0.64

### 3T3 fibroblast-like cell viability

**Figure 3:**
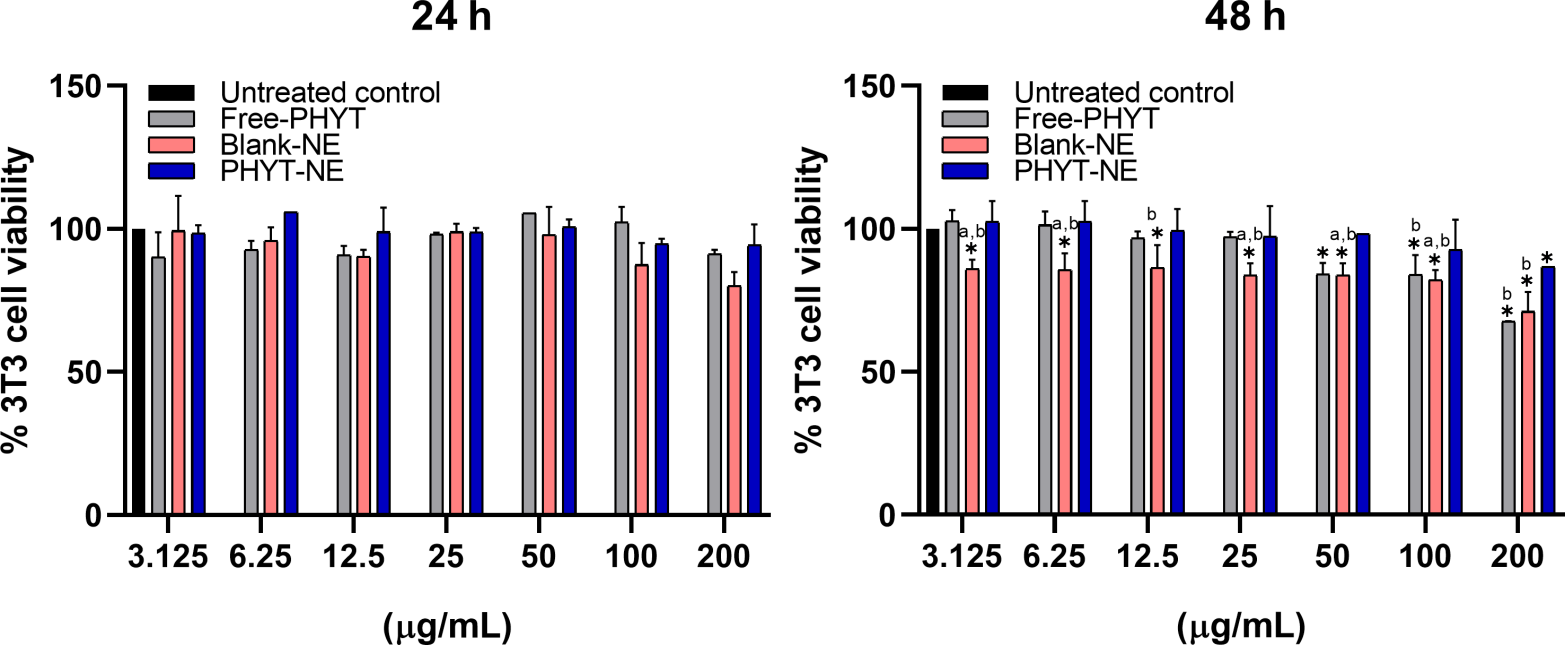
Effects of NEs and free-PHYT treatment on mammalian cells after exposure for 24 and 48 hours. The values represent the mean ± standard deviation of three independent experiments. * Represents significant differences at *p* < 0.05 compared to untreated control; a,b significant differences at *p* < 0.05 between treated groups.

Cell viability in mammalian cells was assessed using 3T3 fibroblast-like cells at 24 and 48 hours (Figure 3). Our results showed that none of the treatments induced significant cytotoxicity in this cell type at 24 hours. However, at 48 hours, cytotoxicity was observed in cells treated with free PHYT and blank-NE at a concentration of 200 µg/mL, resulting in 67% and 71% cell viability, respectively. Interestingly, PHYT-NE remained safe at all tested concentrations and time points, with cell viability above 80% even at the highest concentration.

### In vitro leishmanicidal effects

**Figure 4:**
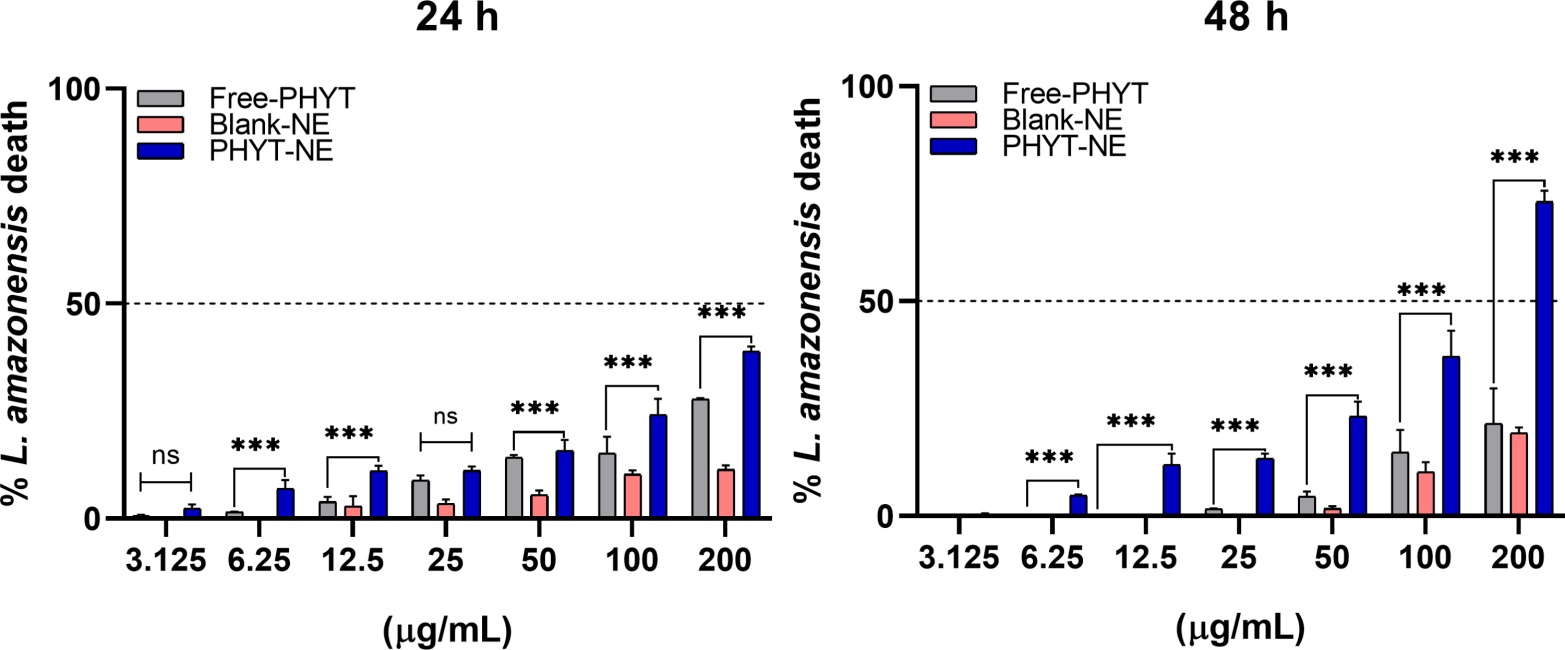
Effect of NEs and free-PHYT on promastigote forms of *L. amazonensis* after exposure for 24 and 48 hours. The values represent the mean ± standard deviation of three independent experiments. *** Represents significant differences at *p* < 0.01.

The leishmanicidal effect of blank-NE, PHYT-NE, and free-PHYT was evaluated against the promastigote forms of *Leishmania amazonensis*. The PHYT-NE exhibited superior efficacy compared to that of free-PHYT at both time points at the same concentrations tested, with estimated IC_50_ values of 289.7 µg/mL at 24 hours and 127.7 µg/mL at 48 hours. In contrast, free-PHYT showed IC_50_ values of 480.5 µg/mL and 713.5 µg/mL at 24 and 48 hours, respectively (Table 2, Figure 4). After 48 hours, the PHYT-NE treatment resulted in approximately 75% of parasite death – nearly double the effect observed after 24 hours. In contrast, the free-PHYT treatment induced less than 30% of parasite death at both 24 and 48 hours (Figure 4).

**Table 2:** IC_50_ values of free-PHYT and PHYT-NE on promastigote forms of *L. amazonensis*.

Samples	IC_50_ 24 h (µg/mL)	IC_50_ 48 h (µg/mL)

free-PHYT	480.5	713.5
PHYT-NE	289.7	127.7

## Discussion

The topical administration of drugs for various diseases offers several advantages over other routes of application, especially in the treatment of cutaneous leishmaniasis [28–29]. To address a promising candidate to this, we developed a stable soybean oil-based nanoemulsion capable of encapsulating PHYT, using the phase inversion composition (PIC) nanoemulsification method. This is a low-shear technique that allows for easy scale-up and helps prevent thermal degradation of sensitive compounds, as well as excessive loss of volatile substances such as PHYT [30].

The small droplet size results in a large surface area, which enables effective interaction with biological membranes and consequently enhances drug penetration and retention [31]. Key formulation parameters – such as drug miscibility with the oil phase, droplet size, and size uniformity – have been directly correlated with permeation efficiency across the skin barrier [32–33]. Specifically, the encapsulation of lipophilic compounds like phytol in finely dispersed droplets within oil-in-water (O/W) nanoemulsions is more effective when the droplets are spherical and have diameters close to 200 nm. This enhanced effect has been attributed to increased Laplace pressure, which refers to the pressure difference between the interior and exterior of a curved interface, and leads to higher surface curvature and increased solute concentration at the droplet interface in the aqueous phase. Such structural features can promote a higher drug flux and localized delivery in the skin layers [34–36].

Chiu et al. (2024) reported a significant increase in curcumin skin permeability when carried by nanoemulsions with a mean diameter between 84.3 and 241.6 nm [37]. In this context, PHYT-NE exhibits suitable droplet size, low PdI, spherical shape, and a pH value within the physiological range of human skin, which suggests that it is a promising formulation for transdermal administration route in the treatment against cutaneous leishmaniasis. Although no permeation assay was conducted in the present study, the physicochemical profile of PHYT-NE supports its potential for efficient skin interaction and warrants further investigation in permeation studies. As a future perspective, ex vivo permeation studies using porcine ear skin or synthetic membranes in Franz diffusion cells could be employed to further evaluate the cutaneous permeation profile of the phytol-loaded nanoemulsion.

In addition to forming nanodroplets, nanoemulsions must maintain their structural and physicochemical integrity over time to ensure consistent performance. In our study, both blank-NE and PHYT-NE remained stable for at least 30 days, with no significant alterations in most evaluated parameters – except the zeta potential – even under centrifugation at different speeds. This behavior can be attributed to the kinetic stabilization achieved through the optimized composition of the surfactant pair, which likely contributed to a robust interfacial film that prevented coalescence and Ostwald ripening [38–40]. Although colloidal stability has been demonstrated, chemical stability studies are still needed to fully confirm the long-term stability and therapeutic reliability of the formulation.

Additionally, the presence of glycerin as a co-solvent may have contributed to the reduction in interfacial tension and improved droplet uniformity, factors known to enhance long-term stability [41]. Importantly, the incorporation of poloxamer 407 increased the viscosity of the external aqueous phase, thereby reducing Brownian motion and droplet collision frequency – mechanisms that are often associated with delayed creaming and phase separation [42–43]. Taken together, these compositional strategies demonstrate the effectiveness of PHYT-NE in maintaining colloidal stability under storage and stress conditions.

For pharmaceutical applications and the intended therapeutic purpose, nanoemulsions must not only be functional but also safe and selective. That is, they should effectively damage the parasite without causing toxicity to host cells – especially intracellular amastigote forms [44].

Previous studies have shown that fibroblasts can act as alternative host cells and have been used to evaluate cytotoxic activity against *Leishmania* spp. in contexts involving epithelial cells and fibroblasts [45]. In addition, reviews on cytotoxicity in cutaneous leishmaniasis highlight the immunological interactions involving multiple cell types beyond macrophages, as well as the role of cytotoxic cells in disease progression and tissue damage, supporting the relevance of studying different cellular models, including fibroblasts [46]. Therefore, the metabolic activity of the fibroblast-like 3T3 cell line was assessed following treatment with the NEs to predict their cytocompatibility and, consequently, their safety. Similarly, promastigote forms of *Leishmania amazonensis* were subjected to the same treatments and conditions to evaluate the leishmanicidal potential of these nanoemulsions.

As PHYT-NE concentrations and exposure times increased, the leishmanicidal activity also improved. Importantly, this formulation did not induce a reduction in cell viability sufficient to indicate cytotoxicity to 3T3 cells, even at the highest concentration and longest exposure time tested. In contrast, this same outcome was not observed with free-PHYT. When both host cells and parasites were treated with free-PHYT, cytotoxicity to fibroblasts was observed at the highest concentration and time point, without a corresponding increase in efficacy against *L. amazonensis* promastigotes compared to the 24-hour exposure. Altogether, these results indicate that incorporating PHYT into the nanoemulsion enhances the selectivity of the molecule, potentiating its therapeutic effect while reducing its toxicity toward mammalian cells. This is supported by a 1.65-fold decrease in the PHYT-NE IC_50_ values at 24 hours, and approximately a 5.6-fold decrease at 48 hours when compared to free-PHYT.

A study conducted by da Silva et al. (2015) [17] demonstrated that the leishmanicidal activity of phytol is associated with induction of mitochondrial membrane depolarization, consequently leading to the generation of reactive oxygen species (ROS) and oxidative stress in promastigotes. It is noteworthy that *Leishmania*, like other trypanosomatids, possesses a single mitochondrion, which is responsible for multiple essential metabolic processes and is crucial for parasite survival; therefore, to exert its effect, the compound must be taken up and accumulate in the cytosol [47]. In the free-living promastigote form, nanoemulsions may facilitate drug access to the intracellular target by more efficiently permeating the membrane of the parasite – either due to their nanoscale size, lipid fusion with membrane components, or disruption of the plasma membrane structure [48–49].

However, several studies have also highlighted the remarkable potential of nanoemulsions against intracellular amastigote forms. In addition to enhancing cutaneous penetration, nanoemulsions also can improve the cellular uptake of various drugs and can facilitate targeted delivery to the intracellular parasites [50–51]. Since the amastigote forms of *Leishmania* spp. reside within the phagolysosomes of macrophages, the phagocytic uptake of nanodroplets significantly increases the intracellular concentration of the drug, thereby enhancing its leishmanicidal efficacy [1,52].

For instance, Mousavi and collaborators (2022) [53] reported that a nanoemulsion loaded with resveratrol effectively inhibited both promastigote and amastigote forms of *Leishmania major*, with an IC_50_ value 2.32-fold lower than that observed for free resveratrol. Similarly, a study conducted by Nahanji et al. (2024) [54] achieved comparable success using fluconazole-loaded NEs against *L. major*, demonstrating a 3-fold and 9-fold reduction in IC_50_ for the promastigote and amastigote forms, respectively, compared to that of free fluconazole. More recently, Cunha et al. (2024) [55] developed NEs containing amphotericin B and paromomycin, and upon evaluating their efficacy against *L. amazonensis* amastigotes, observed that nanoemulsification did not enhance the activity of amphotericin B. In contrast, paromomycin-loaded NEs outperformed the free drug.

All of these studies are in agreement with our results and further underscore the potential of nanoemulsions as effective carriers for antileishmanial agents. Although promastigotes were employed as a proof of concept in this study, it is reasonable to propose that a similar efficacy may also be expected against amastigotes, given the comparable cellular biology and mitochondrial activity between the two stages. The major challenge, however, is the intracellular localization of amastigotes, which requires not only uptake of the nanoemulsion by macrophages but also subsequent internalization by the parasite. Importantly, previous studies have demonstrated that nanoemulsions are efficiently internalized by macrophages, which reinforces the potential of our formulation to also target intracellular amastigotes. Taken together, our results pave the way for further in vitro assays on amastigotes forms, in vivo validation, and clinical translation of PHYT-NE as a safe and effective topical therapy for cutaneous leishmaniasis.

## Conclusion

This is the first time that a soybean oil-based nanoemulsion containing phytol with antileishmanial potential has been reported. The results of this study demonstrate that the phytol-loaded nanoemulsion, developed through a low-energy PIC method, is a stable and physicochemical suitable system for topical and transdermal administration in the future. The PHYT-NE exhibited enhanced leishmanicidal activity against *Leishmania amazonensis* promastigotes in a time- and concentration-dependent manner, while also showing reduced cytotoxicity toward mammalian cells when compared to that of free phytol.

Further studies are still required to strongly support our hypotheses, including evaluation against amastigote forms, safety in macrophages, and confirmation of skin permeation. Nevertheless, the present findings indicate that entrapment enhances selectivity and therapeutic efficacy of phytol. Therefore, PHYT-NE emerges as a promising and safe alternative for the treatment of cutaneous leishmaniasis, warranting additional evaluations to access the scalability potential and in vivo investigations to confirm its efficacy, tissue distribution, and mechanism of action.

## Experimental

### Material

ʟ-α-Phosphatidylcholine (95%) (Avanti Polar lipids, United States); polaxamer 407 (Sigma-Aldrich, Brazil); glycerin (Vetec, Brazil); phytol (97%), mixture of isomers (Sigma-Aldrich, Brazil); soybean oil (Sigma-Aldrich, Brazil); Tween^®^ 80 (Sigma-Aldrich, Brazil); purified water (obtained from a reverse osmosis purification equipment, model OS50 LX, Gehaka, Brazil) were used for nanoemulsion preparation. Mammalian cells were cultured in DMEM, High Glucose (Life Technologies, Cat. 12800-058, Bleiswijk, The Netherlands) supplemented with 10% fetal bovine serum (FBS, Life Technologies, Cat. 12657029, Bleiswijk, The Netherlands) and maintained in an atmosphere of 5% CO_2_ at 37 °C. Cell viability was assessed using MTT reagent (Sigma-Aldrich, Cat. M5655, St. Louis, MO, USA), with formazan solubilization in ethanol (Sigma-Aldrich, Brazil) when required. In vitro antileishmanial activity was performed in RPMI 1640 medium (Sigma-Aldrich, Brazil) supplemented with FBS and streptomycin antibiotic 100 IU/mL, with parasite viability measured using resazurin solution (Sigma-Aldrich, Brazil).

### Nanoemulsion preparation and composition

Nanoemulsions were prepared using the phase inversion composition method, a low-energy technique wherein the aqueous phase (AP) was gradually added dropwise to the oil phase (OP) under continuous magnetic stirring at 1500 rpm, using a magnetically stirrer (IKA^®^ C-MAG HS7) at a temperature of 25 ± 2 °C. Following the complete addition of the AP, the mixtures were stirred for an additional 30 min to ensure homogeneity. The AP consisted of purified water and poloxamer 407 (POL), as a stabilizing agent, while the OP was composed of soybean oil (SO), glycerin, as a co-solvent, Tween^®^ 80, and ʟ-α-phosphatidylcholine (PC), as surfactants. For nanoemulsions containing phytol, 10 mg/g of the drug was added to OP. Subsequent to the nanoemulsification step, the nanoemulsions were transferred into hermetically sealed glass vials and stored at room temperature for further analysis. All samples were prepared in triplicate and data were expressed as mean ± standard deviation. The NEs composition was described in Table 3.

**Table 3:** Composition of the nanoemulsion (per 10 g).

	SO (g)	Tween^®^ 80 (g)	PC (g)	Glycerin (g)	POL 407 (g)	PHYT (g)	Water (mL)

blank-NE	0.5	0.4	0.1	1.0	1.0	–	7.0
PHYT-NE	0.5	0.4	0.1	1.0	1.0	0.1	6.9

### Physicochemical characterization

#### Droplet size, polydispersity index and zeta potential evaluation

The droplet size was determined by dynamic light scattering using a Zetasizer Nano ZS instrument (Malvern Instruments, UK) at 25 ± 2 °C, with a detection wavelength of 633 nm and a backscattering angle of 173°. The zeta potential was assessed via electrophoretic mobility measurements using the same equipment under identical temperature conditions. For both analyses, samples were appropriately diluted at a 1:100 (v/v) ratio.

#### Assessment of hydrogenic potential (pH)

The pH value was determined by the potentiometric method by inserting the electrode (Digimed, mod. DM-22) directly into the samples. In this assay, the NE were used in triplicates at room temperature (24 ± 2 °C).

#### Colloidal and physicochemical stability

For accelerated stability analysis, 1 mL aliquots of the nanoemulsion were transferred to Eppendorf tubes and subjected to three centrifugation cycles at 960 and 8600*g* for 15 min, each using a mini centrifuge (Fisherbrand^®^, model Gusto, Illinois, USA), following a protocol adapted from Saberi et al. (2013) [41]. After centrifugation, samples were analyzed regarding droplet size, PdI, and ZP. All measurements were performed in triplicates at room temperature (25 ± 2 °C).

In addition, colloidal stability of non-centrifuged nanoemulsions was evaluated over a 30-day period by monitoring droplet size, PdI, ZP, and pH using the same methodologies previously described.

#### Morphology

The morphology of the nanoemulsion droplets was examined by transmission electron microscopy using a FEI Tecnai G2 Spirit Biotwin microscope operating at 120 kV (FEI Company, Hillsboro, OR, USA). The samples were diluted in purified water at a 1:20 (v/v) ratio, and a drop of the diluted suspension was deposited onto square-mesh copper grids and allowed to adsorb for 2 min. The grids were then air-dried at room temperature prior to imaging.

### Cytocompatibility

#### Mammalian cell culture

The mouse embryonic fibroblast cell line 3T3 cells (ATCC^®^ CRL-1658) were cultured in 25 cm^2^ culture flasks with Dulbecco's Modified Eagle Culture Medium (DMEM, Life Technologies, Cat. 12800-058, Bleiswijk, The Netherlands), supplemented with 10% fetal bovine serum (FBS, Life Technologies, Cat. 12657029, Bleiswijk, The Netherlands), remaining in an atmosphere of 5% CO_2_ at 37 °C.

#### Cell viability assay

The 3T3 cells maintained in culture were treated with trypsin, centrifuged at 200*g* for 5 min, resuspended in DMEM, and seeded at a concentration of 5 × 10^4^ cells/mL in 96-well microplates and incubated for 24 h at 37 °C and atmosphere at 5% CO_2_. Subsequently, the cells were deprived of serum for 24 h with serum-free DMEM. After the deprivation period, cells were treated with the test formulations diluted in DMEM containing 10% FBS to restore normal growth conditions. The nanoemulsions were filtered through a 0.45 µm Millipore filter, and drug solutions were filtered through a 0.22 µm Millipore filter. Then, cells were treated with blank nanoemulsions and PHYT in serial dilutions, considering the concentration of drugs in the formulations (200 to 3.125 µg/mL) and incubated for 24 and 48 h. These incubation times were selected to evaluate the acute response, focusing on immediate effects.

After this period, the supernatant was removed, and 100 μL of an MTT solution in DMEM (final concentration 1 mg/mL) was added to each well. The plates were incubated at 37 °C with 5% CO_2_ for 4 h. After this time, the MTT solution was removed, and 100 μL of ethanol was added to each well. The plates were protected from light and agitated for 20 min. The absorbance was measured at 570 nm using an ELISA microplate reader (Biotek^®^ Epoch), and the obtained values were applied following Equation 1. Cells grown under the same conditions and that did not receive treatment with the systems were used as negative control.


[1] $$\%\text{cell viability}=\frac{{\text{Abs}}_{\text{sample}}}{{\text{Abs}}_{\text{control}}}\times 100,$$

where Abs_sample_ is the absorbance of cells treated with the sample and Abs_control_ is the absorbance of untreated control cells. The statistical analysis performed in the in vitro cytocompatibility assay was the two-way ANOVA, followed by Tukey’s post hoc test.

### In vitro antileishmanial activity

#### Parasite cell culture

The cultures of *Leishmania amazonensis* promastigotes were carried out in 25 cm^2^ flasks containing RPMI medium (Sigma-Aldrich, Brazil) supplemented with 10% FBS and 10% streptomycin antibiotic (100 IU/mL) at 27 °C. For the tests, cultures were used after the 4th day of supplementation, obtaining parasites at the end of the log phase, that is, with the greatest possible growth potential.

#### Antiparasitic activity of nanoemulsions on *Leishmania amazonensis* promastigotes

To assess the antiparasitic activity, *Leishmania amazonensis* promastigotes after the 4th days of growing in RPMI medium were counted using a Neubauer chamber, diluted in RPMI medium supplemented with 10% FBS and 10% streptomycin antibiotic (100 IU/mL) at 27 °C, and adjusted to a concentration of 1 × 10^7^ parasites/mL. Afterward, the aliquots of 200 µL were dispensed in 96‑well plates in triplicates and treated with different concentrations of free-PHYT, blank-NE, and PHYT-NE (200 to 3.125 µg/mL); moreover, amphotericin B (2.5 µg/mL) was used as the positive control (100% death). After 24 and 48 h of exposure at 26 °C, the parasite viability was determined by the resazurin reduction assay. For that, 20 µL of 3 mM resazurin solution (Sigma-Aldrich, Brazil) was added to each well and plates were incubated for 24 h. The absorbance was read at 570 and 600 nm (Epoch, BioTek). The results of antiparasitic activity were determinate by Equation 2:


[2] $$\%\text{inhibition}=100-\left[\frac{\text{A}570\text{t}-(\text{A}600\text{t}\times \text{R}0)}{\text{A}570\text{c}-(\text{A}600\text{c}\times \text{R}0)}\times 100\right],$$

where A570t = absorbance of the treatment at a wavelength of 570 nm; A600t = absorbance of the treatment at a wavelength of 600 nm; A570c = absorbance of the control at a wavelength of 570 nm; A600c = absorbance of the control at a wavelength of 600 nm; R0 = correction factor of the medium interacting with resazurin, obtained using the following formula: R0 = C_medium_570nm/C_medium_600nm, with C_medium_570nm = absorbance of the medium at a wavelength of 570 nm and C_medium_600nm = absorbance of the medium at a wavelength of 600 nm.

#### Statistical analysis

Samples were prepared and analyzed in triplicates. Results were expressed as mean ± standard deviation. Initially, the normality test was performed, followed by Student's t test for paired analysis of two or one-way analysis of variance (ANOVA) when applicable, using the GraphPad Prism 8 software. The results were considered statistically significant for *p* < 0.05.

## Supporting Information

File 1 Additional figure.

## Data Availability

All data that supports the findings of this study is available in the published article and/or the supporting information of this article.
