# Supplementary material for: Phytol-loaded soybean oil nanoemulsion as a promising alternative against Leishmania amazonensis
Source: Beilstein J Nanotechnol. 2025 Oct 21;16:1826–36. doi: 10.3762/bjnano.16.126 (PMC12557441; doi:10.3762/bjnano.16.126)
Supplement: File 1 — Additional figure. [file Beilstein_J_Nanotechnol-16-1826-s001.pdf]

BEILSTEIN JOURNAL OF NANOTECHNOLOGY

# Supporting Information

for

# Phytol-loaded soybean oil nanoemulsion as a promising alternative against *Leishmania amazonensis*

Victória Louise Pinto Freire, Mariana Farias Alves-Silva, Johny W. de Freitas Oliveira, Matheus de Freitas Fernandes-Pedrosa, Alianda Maira Cornélio, Marcelo de Souza-Silva, Thayse Silva Medeiros and Arnóbio Antônio da Silva Junior

*Beilstein J. Nanotechnol.* **2025,** *16,* 1826–1836. doi:10.3762/bjnano.16.126

## Additional figure

License and Terms: This is a supporting information file under the terms of the Creative Commons Attribution License (https://creativecommons.org/licenses/by/4.0). Please note that the reuse, redistribution and reproduction in particular requires that the author(s) and source are credited and that individual graphics may be subject to special legal provisions.
The license is subject to the *Beilstein Journal of Nanotechnology* terms and conditions: (https://www.beilstein-journals.org/bjnano/terms)

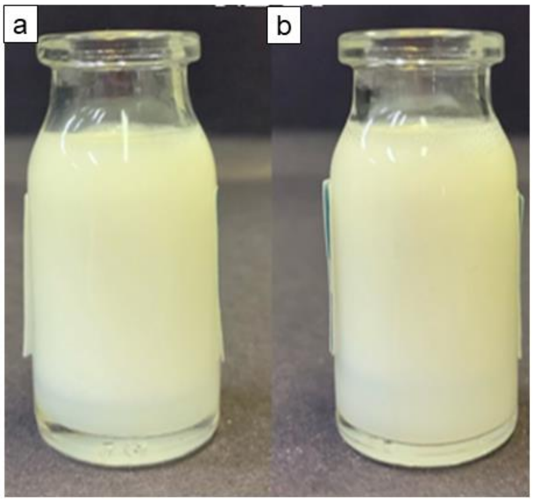


**Figure S1:** Macroscopic appearance of the nanoemulsions after 24 h of preparation. (a) Blank-NE; (b) PHYT-NE.
